# Second trimester maternal serum D-dimer combined with alpha-fetoprotein and free β-subunit of human chorionic gonadotropin predict hypertensive disorders of pregnancy: a systematic review and retrospective case–control study

**DOI:** 10.1186/s12967-021-02718-4

**Published:** 2021-03-02

**Authors:** Yiming Chen, Yijie Chen, Xue Wang, Xuelian Chu, Wenwen Ning, Linyuan Gu, Liyao Li, Zhen Xie, Caihe Wen

**Affiliations:** 1 Department of Prenatal Diagnosis and Screening Center, Hangzhou Women’s Hospital (Hangzhou Maternity and Child Health Care Hospital), No. 369, Kunpeng Road, Shangcheng District, Hangzhou, 310008 Zhejiang China; 2 Department of the Fourth Clinical Medical College, Zhejiang Chinese Medical University, Hangzhou, 310053 Zhejiang China; 3 Nanjing Medical University, Nanjing, 210029 Jiangsu China; 4 Department of Laboratory, Maternal and Child Health Hospital of Yuhang District, Hangzhou, 311100 Zhejiang China; 5 Department of Obstetrics, Hangzhou Women’s Hospital (Hangzhou Maternal and Child Health Care Hospital), Hangzhou, 310008 Zhejiang China

**Keywords:** D-dimer, Alpha-fetoprotein, Free β subunit of human chorionic gonadotropin, Hypertensive disorders of pregnancy, Gestational hypertension, Preeclampsia

## Abstract

**Background:**

This study investigated whether maternal serum D-dimer (DD) alone or DD combined with alpha-fetoprotein (AFP) and free β-subunit of human chorionic gonadotropin (free β-hCG) in the second trimester could be used to predict hypertensive disorders of pregnancy (HDP).

**Materials and methods:**

In this retrospective case–control study, the data of gravidas patients who delivered at hospital were divided into the following groups: control (n = 136), gestational hypertension (GH, n = 126), preeclampsia (PE, n = 53), and severe preeclampsia (SPE, n = 41). Receiver operator characteristic (ROC) curves were used to evaluate the diagnostic value of maternal serum DD, AFP, and free β-hCG levels for HDP.

**Results:**

DD levels of the GH, PE, and SPE groups were significantly higher than that of the control group (*P* < 0.001). The order of effectiveness for models predicting HDP was as follows: DD + AFP + free β-hCG > DD > DD + AFP > DD + free β-hCG > AFP + free β-hCG > AFP > free β-hCG. For predicting different types of HDP, DD alone had the best diagnostic value for SPE, followed by PE and GH. DD alone had a sensitivity of 100% with a 0% false negative rate and had the highest positive likelihood ratio (+ LR) for SPE. DD alone in combination with AFP alone, free β-hCG alone and AFP + free β-hCG could reduce false positive rate and improve + LR.

**Conclusion:**

DD is possible the best individual predictive marker for predicting HDP. Levels of DD alone in the second trimester were positively correlated with the progression of elevated blood pressure in the third trimester, demonstrating the predicting the occurrence of HDP. The risk calculation model constructed with DD + free β-hCG + AFP had the greatest diagnostic value for SPE.

## Background

Hypertensive disorders of pregnancy (HDP) are obstetric complications that can lead to adverse events for both the pregnant woman and the fetus, including increased risk of maternal stroke, lower birth weight, neonatal intensive care needs, and maternal and perinatal deaths [1–3]. HDP complicate up to 10% of pregnancies worldwide, seriously threatening pregnant women and perinatal children, and are the second leading cause of maternal death and amniotic fluid embolism, but their etiology and pathogenesis are not fully clear [4, 5]. Preeclampsia (PE) is the most common pregnancy complication associated with HDP, affecting 5–8% of all pregnancies. Peripheral vasoconstriction secondary to maternal systemic inflammation is sufficient for the development of PE-induced hypertension [6, 7]. The identification of HDP during pregnancy plays a vital role in first-line management and appropriate referrals to specialist care [6]. Furthermore, HDP are known to predispose women to ongoing hypertension and associated cardiovascular morbidity later in life [8].

Noninvasive examination of maternal serum alpha-fetoprotein (AFP) and free β subunit of human chorionic gonadotropin (free β-hCG) have been extensively applied in the detection of fetal Down’s syndrome (DS), open neural tube defects, Edwards’ syndrome, and other diseases [9, 10]. One meta-analysis showed that combined screening markers detected approximately 90% of all DS pregnancies, with a false positive rate of 5% [11].

D-dimer (DD) is a small protein fragment that is degraded by fibrinolysis. It is the smallest fragment of the fibrin degradation products and increases significantly during pregnancy [12, 13]. DD is one of the most valuable indicators for the diagnosis and management of thrombotic states in those with venous thromboembolism, atrial fibrillation, and cancer, as well as those who are pregnant or undergoing surgery. DD has high sensitivity and negative predictive value (NPV), but its specificity is lower [14, 15]. A case–control study showed that elevated levels of DD measured at gestational ages above 20 weeks were significantly associated with severe preeclampsia (SPE) [16]. This research revealed the pathophysiological mechanisms that contributed to the enhanced coagulation and impaired fibrinolysis associated with the condition [16, 17]. Our previous research indicated that elevated maternal serum AFP MoM values in the second trimester were a risk factor for PE, but AFP alone was less sensitive and reliable than combined screening markers [18]. Bredaki et al. showed that measuring maternal serum AFP in the first and second trimesters improved the prediction of preterm PE [19]. There are some markers detected in second trimester for predicting HDP. Expression of placental growth factor (PlGF) precede preeclampsia by several weeks and its expression decreases in preeclampsia. Recent research suggests that PlGF may be a useful adjunct in the management of pre-eclampsia [20]. The uterine artery doppler acquisition is in the prediction of subsequent development of PE and improve risk assessment [21].

To our knowledge, there has been little research on the prediction of HDP using a combination of DD, AFP, and free β-hCG in the second trimester. Currently, we have no method to accurately predict who early on in pregnancy will go on to develop HDP after 20 weeks gestation, missing a potential window for early intervention and prevention of hypertension-related complications. The objectives of this study were to present the distributions of different screening marker concentrations and examine their predictive value for different types of HDP.

## Methods

### Study design

This was a retrospective observational case–control study of women recruited at their routine second-trimester aneuploidy screening appointment (15 weeks to 20 weeks and 6 days of gestation) at Hangzhou Women’s Hospital (Hangzhou Maternity and Child Health Care Hospital) between November 2014 and April 2019. Finally, we recruited 356 pregnant women, including 136 HDP-free pregnant women and 220 pregnant women with HDP (126 cases of gestational hypertension [GH], 53 cases of PE, and 41 cases of SPE) from whom we obtained second trimester serum samples. We aimed to recruit similar numbers of PE and SPE patients and randomly selected participants for the control group. This study was approved by the medical ethics committee of the Hangzhou Women’s Hospital [2020] Medical Ethics Review A (10)-11.

### Diagnostic criteria and exclusion criteria

**Fig. 1:**
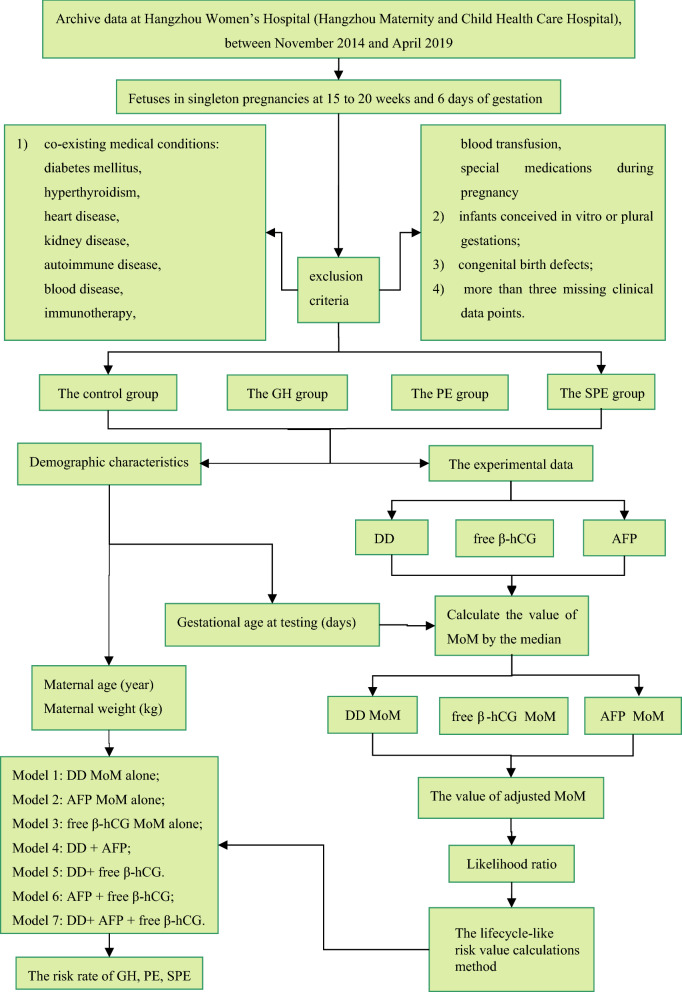
Workflow of development of prediction model

After careful screening, a total of 356 pregnant women with a complete set of available serum samples were selected. Patients were divided into four groups (control, GH, PE, and SPE) based on the Guidelines for Diagnosis and Treatment of Hypertension during Pregnancy (2015) [22]. GH was defined as new-onset hypertension (systolic blood pressure ≥ 140 mmHg and/or diastolic blood pressure ≥ 90 mmHg, 1 mmHg = 0.133 kPa) after 20 weeks of gestation with recovery within 12 weeks after delivery and negative urine protein. Patients diagnosed with PE had blood pressure levels higher than 140/90 mmHg and proteinuria but had no other indicators. Patients diagnosed with SPE met one or more criteria (hypertensive crisis, renal or hepatic dysfunction, pulmonary edema, or eclampsia) at hospital admission or during hospitalization. The exclusion criteria were as follows in Fig. 1: (1) co-existing medical conditions, such as diabetes mellitus, hyperthyroidism, heart disease, kidney disease, autoimmune disease, blood disease, or a history of immunotherapy, blood transfusion, or special medications during pregnancy; (2) history of special pregnancy, such as infants conceived in vitro or plural gestations; (3) congenital birth defects; or (4) more than three missing clinical data points.

### Reagents and apparatus

Enzyme linked immunosorbent assays (ELISAs) were used to detect DD (RK72S34, IBM, San Francisco, USA). Plates were washed with a 988 Plate Washer (Tianshi, Beijing, China) and detected using an Rt-6100 Plate Reader (Rayto, Shenzhen, China). A1235 Automatic Immunoassay System (PerkinElmer, Shelton, CT, USA) and a double labeling kit (AFP/free β-hCG) for the second trimester were used with washing liquid, quality control samples, and a range of standards (Wallac Oy, Turku, Finland).

### Blood samples and screening indicators

Fasting venous blood samples (2–3 mL) were collected from pregnant women between 15 and 20 weeks and 6 days of gestation. After detection of AFP and free β-hCG, the remaining serum was stored at − 80 °C. After accumulating a certain number of specimens, the information of the HDP and control groups was matched with the stored serum samples before measuring DD. The DD tests correlated between fresh and frozen sample. The acceptable freeze–thaw cycles for DD at − 80 °C [23].

### Construction of risk prediction models for HDP

A lifecycle-like risk value calculation model for HDP consisting of a MoM calculation, a lifecycle-like risk value calculation, and a risk definition was constructed.

#### MoM calculation

In unaffected pregnancies, aneuploidy screening markers change with gestational age [24]. Therefore, levels of DD, AFP and free β-hCG were expressed using MoM values. MoM values are calculated by dividing the original concentrations of DD, AFP and free β-hCG by the population median at the gestational age at which the marker was tested. MoM adjustments for covariables such as gestational age and maternal weight were also performed according to published data [24].

The MoM value calculation formula: $MoM = \frac{Original\;Conj.}{{Median\  Original\ Conj}}$.

The MoM value adjusted by maternal weight and gestational age (GA):

$${{GA}_{Med}} = 10^{(-2.577\times{GA}+0.004083\times{{GA}^{2}}+0.004083\times{{GA}^{3}}-0.0001541\times{{GA}^{4}}-21.36)}$$

Same as maternal weight.

$$Adjusted\ MoM = \frac{MoM}{{{\text{Gestational age}}\ Median \times Maternal \ Weight\ Median}}$$

#### Lifecycle-like risk value calculations

Risk models were constructed using DD, AFP and free β-hCG alone and in combination using the likelihood ratio construction method. The MoM values of AFP, free β-hCG, and DD had multivariate normal distributions. Parameters corresponding to marker distributions were calculated according to the calculated risk, the modeling method used, and the distribution. Seven HDP risk predictive models were constructed using the likelihood for the distribution, as shown in Fig. 1: model 1, DD MoM alone; model 2, AFP MoM alone; model 3, free β-hCG MoM alone; model 4, DD + AFP; model 5, DD + free β-hCG; model 6, AFP + free β-hCG; model 7, DD + AFP + free β-hCG.

#### Risk definition

We defined an equation for each marker’s median specificity from a preliminary study [25]

using the likelihood ratio calculation:

$${LR \, }_{multinorm}=\frac{\mathrm{likelihood\,of\,SPE\,group}}{\mathrm{likelihood\,of\,control\,group}}$$

The likelihood for a three-dimensional normal distribution was calculated as follows:

Let *χ* be the vector of three-dimension normal distribution: *χ* = (*χ*_1_, *χ*_2_, *χ*_3_)^T^

$$f\left( x \right) = \frac{{1}}{{\sqrt {\left( {{2}\pi } \right)^{{3}} \cdot {|}\Sigma {|}} }}\exp \left( { - \frac{1}{{2}}\left[ {\left( {x - \mu } \right)^{\rm T} \cdot \Sigma^{ - 1} \cdot \left( {x - \mu } \right)} \right]} \right)$$

where, |Σ| is the determinant of the covariance matrix of *χ*, Σ^−1^ represents the inverse matrix of the covariance matrix of *χ*, and $\mu$ is the average. *χ* denotes the logarithm of the DD MoM, AFP MoM, and free β-hCG MoM value [24].

The ultimate risk of SPE was calculated as follows:

$$risk_{SPE} = \frac{1}{{LR_{multinorm} \times risk_{Maternal \ age} }}$$

The risk models for GH, PE and HDP were constructed using the same method.

### Statistical analysis

Microsoft Excel (2007) software was used to build a database, and IBM SPSS 21.0 Statistics version 21.0 (IBM Corp., Armonk, NY, USA) was used for statistical processing. One-sample Kolmogorov–Smirnov tests were used to test normality. Maternal age, gestational age at testing, and maternal weight in the second trimester were normally distributed and expressed as mean ± standard deviation ($\overline{x}$ ± s). One-way analysis of variance was employed to test differences between groups and Dunnett T tests were employed to test differences among groups. DD, AFP, and free β-hCG showed a skewed distribution and are expressed as median and percentile [M (P_2.5_, P_97.5_)]. Skewed data were compared within or between groups using the Kruskal–Wallis H (K) test or the Mann–Whitney U test. Risk models were constructed using the likelihood ratio construction method with Python 3.7 software (https://www.python.org/). Cut-off and area under the curve (AUC) values were determined using receiver operator characteristics (ROC) curves to assess the diagnostic value of DD, AFP, and free β-hCG for HDP, and the optimal cut-off and AUC values and Youden index were calculated. The positive predictive value (PPV), NPV, positive likelihood ratio (+ LR) and negative likelihood ratio (−LR) values were used to assess model performance. *P* < 0.05 was considered statistically significant.

## Results

### Comparison of basic demographic data

The characteristics of the study participants are summarized in Table 1. The maternal age of the GH, PE and SPE groups was higher than that of the control group, and there was no statistical difference between the groups (*F* = 1.055, *P* = 0.368). The second trimester gestational age of the GH, PE and SPE groups was lower than that of the control group, and there was no statistical difference between groups (*F* = 1.460, *P* = 0.225). The second trimester maternal weight of the GH, PE and SPE groups was higher than that of the control group and the difference was statistically significant (*F* = 23.809, *P* < 0.001).

**Table 1:** Comparison of demographic data in the second trimester between the different groups

Groups	n	Maternal age (years)	Gestational age at testing (days)	Maternal weight (kg)
Control	136	28.26 ± 2.67	118.00 ± 4.00	53.64 ± 5.41
GH	126	28.84 ± 3.35	117.00 ± 5.00	63.06 ± 11.35
PE	53	28.86 ± 2.98	118.00 ± 6.00	59.68 ± 11.26
SPE	41	28.83 ± 3.05	118.00 ± 4.00	57.02 ± 8.32
*P*		0.368	0.225	< 0.001*

*GH* gestational hypertension, *PE* preeclampsia, *SPE* severe preeclampsia.*P < 0.001

### Comparison of maternal serum AFP, free β-hCG, and DD across the four groups (Table 2 and Fig. 2)

**Table 2:** AFP, free β-hCG and DD (MoM) in the different groups at the screening

Groups	n	DD MoM	AFP MoM	Free β-hCG MoM
Control	136	0.89 (0.50–1.42)	0.96 (0.58–1.68)	1.02 (0.36–3.24)
GH	126	1.02 (0.77–1.23)	0.98 (0.58–1.65)	0.93 (0.40–2.71)
PE	53	1.16 (0.69–1.47)	0.99 (0.47–1.62)	0.97 (0.24–2.80)
SPE	41	1.34 (1.06–1.64)	1.09 (0.39–2.23)	1.07 (0.43–3.52)
*P*		< 0.001*	0.009**	0.309

*DD* D-dimer, *AFP* alpha-fetoprotein, *free β-hCG* free β subunit of human chorionic gonadotropin, *GH* gestational hypertension, *PE* preeclampsia, *SPE* severe preeclampsia, *MoM* multiple of the median.*P < 0.001; **P < 0.05

**Fig. 2:**
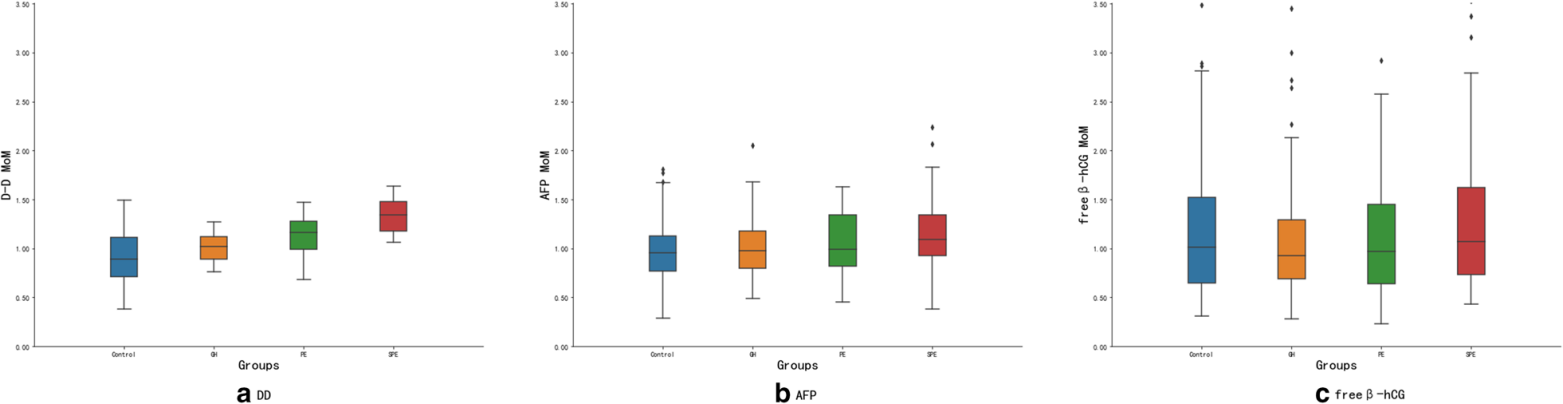
Comparison of DD, AFP, and free β-hCG MoM among the four groups. **a** DD MoM; **b** AFP MoM; **c** free β-hCG MoM. *DD* D-dimer, *AFP* alpha-fetoprotein, *free β-hCG* Free beta subumit of human chorionic gonadotropin, *GH* gestational hypertension; *PE* Preeclampsia, *SPE* severe preeclampsia

DD levels differed significantly between the groups and pairwise comparisons between groups showed statistically significant differences (*χ*^*2*^ = 104.427, *P* < 0.001). The MoM value for DD of the SPE groups was 1.34 and was significantly higher than the MoM of the control, GH and PE group (0.89, Z = 3.034; 1.02, Z = 6.053; 1.16, Z = 9.398, all *P* < 0.05). The level of maternal serum DD tended to gradually increase with the progress of hypertension, as shown in Fig. 2a.

The MoM values for AFP of the GH, PE, and SPE groups were 0.98, 0.99 and 1.09 respectively, and were significantly higher than that of the control group (0.96; *χ*^*2*^ = 11.691, *P* = 0.009). In addition, AFP levels of the SPE group were higher than those of the control groups (*Z* = 3.336, both *P* = 0.005), but AFP levels were not statistically different between the other groups (*P* > 0.05). (Fig. 2b).

The MoM values for free β-hCG of the GH, PE, and SPE groups were 0.93, 0.97 and 1.07 respectively, which was lower than that of the control group 1.02, but there was no statistical difference between the other groups (*χ*^*2*^ = 3.593, *P* = 0.309), as shown in Fig. 2c.

### Predictive value of DD, AFP, and free β-hCG for GH, PE, SPE, and HDP (Table3 and Fig. 3)

**Table 3:** Predicting value of DD, AFP and free β-hCG separate or joint screening for GH, PE, SPE and HDP

Groups (n) screening indicators	AUC	95%CI	*P*	Cut-off	Sensitivity	Specificity	Youden index
GH (126)							
DD	0.642	0.573–0.711	< 0.001*	0.755	1.000	0.324	0.324
AFP	0.537	0.467–0.608	0.295	0.875	0.667	0.426	0.093
Free β-hCG	0.537	0.467–0.608	0.295	1.155	0.698	0.449	0.147
DD + AFP	0.746	0.686–0.805	< 0.001*	0.652	0.921	0.500	0.421
DD + free β-hCG	0.743	0.683–0.802	< 0.001*	0.573	0.937	0.500	0.437
AFP + free β-hCG	0.553	0.484–0.623	0.136	1.037	0.651	0.485	0.136
DD + AFP + free β-hCG	0.757	0.700–0.815	< 0.001*	0.790	0.897	0.537	0.434
PE (53)							
DD	0.767	0.698–0.835	< 0.001*	0.965	0.849	0.640	0.489
AFP	0.567	0.471–0.664	0.151	1.205	0.377	0.816	0.194
Free β-hCG	0.511	0.418–0.604	0.814	0.440	0.113	0.971	0.084
DD + AFP	0.765	0.698–0.833	< 0.001*	0.804	0.868	0.618	0.486
DD + free β-hCG	0.772	0.704–0.840	< 0.001*	0.947	0.830	0.647	0.477
AFP + free β-hCG	0.547	0.458–0.637	0.312	0.873	0.792	0.338	0.131
DD + AFP + free β-hCG	0.788	0.723–0.854	< 0.001*	0.952	0.849	0.647	0.496
SPE (41)							
DD	0.905	0.862–0.948	< 0.001*	1.050	1.000	0.713	0.713
AFP	0.675	0.581–0.768	0.001**	0.910	0.854	0.449	0.302
Free β-hCG	0.561	0.462–0.659	0.240	0.570	0.951	0.176	0.128
DD + AFP	0.904	0.861–0.947	< 0.001*	0.543	1.000	0.757	0.757
DD + free β-hCG	0.894	0.848–0.939	< 0.001*	0.810	0.951	0.765	0.716
AFP + free β-hCG	0.693	0.603–0.784	< 0.001*	0.807	0.878	0.441	0.319
DD + AFP + free β-hCG	0.912	0.897–0.953	< 0.001*	0.706	1.000	0.757	0.757
HDP = GH + PE + SPE (220)							
DD	0.721	0.663–0.779	< 0.001*	0.965	0.736	0.640	0.376
AFP	0.570	0.510–0.631	0.026**	1.105	0.395	0.743	0.138
Free β-hCG	0.513	0.450–0.575	0.684	1.155	0.632	0.449	0.080
DD + AFP	0.719	0.662–0.776	< 0.001*	0.614	0.932	0.404	0.336
DD + free β-hCG	0.717	0.660–0.774	< 0.001*	0.492	0.950	0.397	0.347
AFP + free β-hCG	0.546	0.484–0.607	0.146	0.983	0.668	0.449	0.117
DD + AFP + free β-hCG	0.736	0.679–0.792	< 0.001*	1.429	0.709	0.684	0.393

*DD* D-dimer, *AFP* alpha-fetoprotein, *free β-hCG* free beta subumit of human chorionic gonadotropin, *MoM* multiple of the median, *GH* gestational hypertension, *PE* Preeclampsia, *SPE* severe preeclampsia, *HDP* Hypertensive disorders of pregnancy, *AUC* area under curve. *P < 0.001; **P < 0.05

**Fig. 3:**
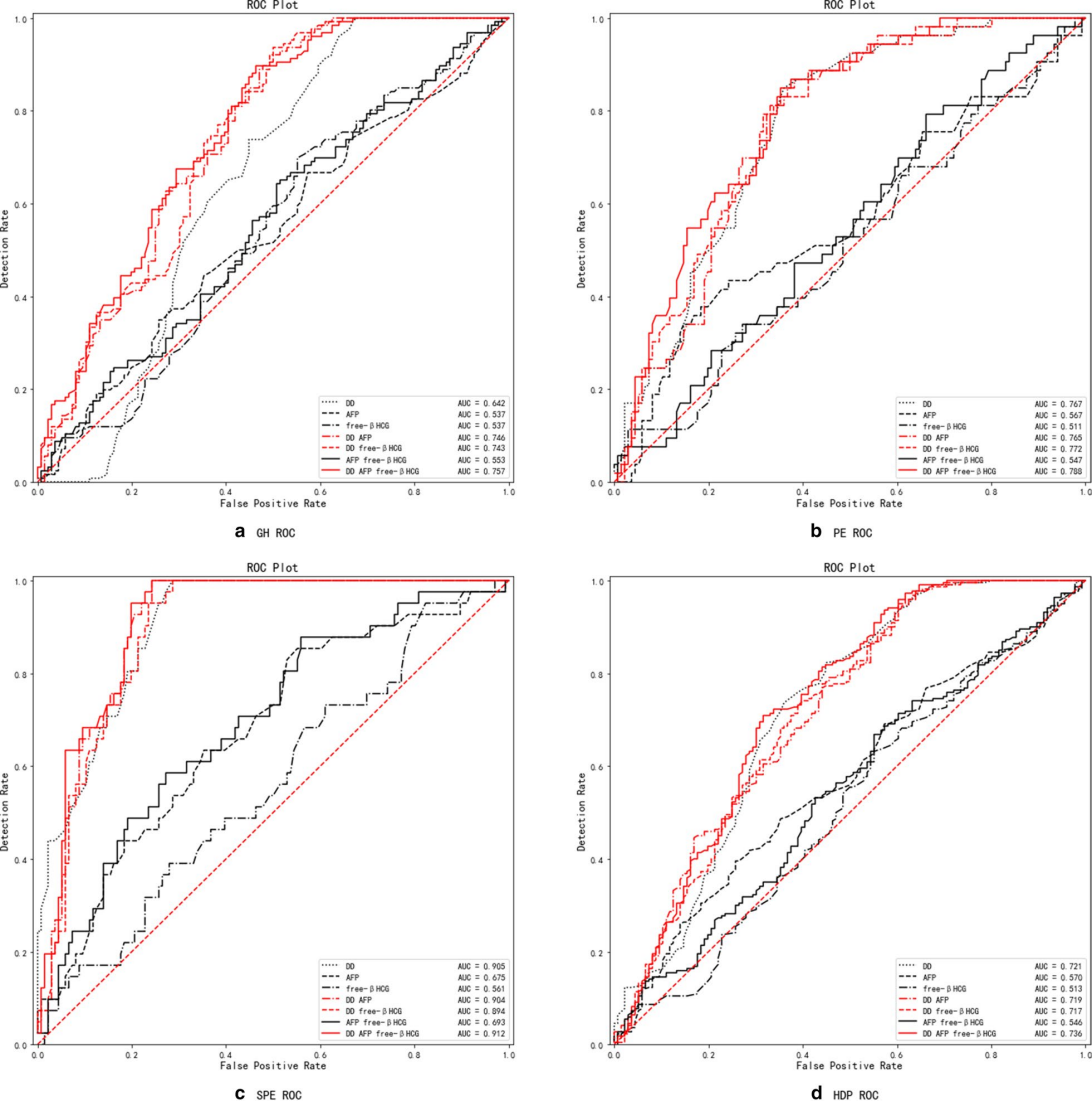
ROC curve for the prediction value of GH, PE, or SPE. **a** ROC curve for the prediction of GH; **b** ROC curve for the prediction of PE; **c** ROC curve for the prediction of SPE; **d** ROC curve for the prediction of HDP (GH + PE + SPE). *DD* D-dimer, *AFP* alpha-fetoprotein, *free β-hCG* free beta subumit of human chorionic gonadotropin, *GH* gestational hypertension, *PE* Preeclampsia, *SPE* severe preeclampsia

For predicting GH and PE, DD alone had diagnostic value, with an AUC of 0.642, 0.767 (*P* < 0.001). DD combined with AFP alone, free β-hCG alone and AFP + free β-hCG had diagnostic value and improved the specificity for GH and PE (*P* < 0.001, Fig. 3a, b). AFP alone, DD alone, DD + AFP, DD + free β-hCG, AFP + free β-hCG and DD + AFP + free β-hCG all had diagnostic value as predictors for SPE (all *P* < 0.05, Fig. 3c). The sensitivities of DD and DD + AFP + free β-hCG were highest, and the sensitivity of DD + AFP + free β-hCG was 1.000. Both DD alone and DD + AFP + free β-hCG were found to have diagnostic value for predicting HDP (*P* < 0.001, Fig. 3d) with the following order of effect: DD + AFP + free β-hCG > DD > DD + AFP > DD + free β-hCG > AFP > AFP + free β-hCG > free β-hCG. Both DD alone, DD + AFP, DD + free β-hCG, and DD + AFP + free β-hCG had diagnostic value for predicting different types of HDP (*P* < 0.001) with the following order of effect: SPE > PE > GH.

### Value evaluation of risk models (Table 4)

**Table 4:** The value evaluation for the risk models

Groups (n) screening indicators	FPR	FNR	PPV	NPV	+ LR	− LR
GH (126)						
DD	0.676	0.000	0.578	1.000	1.478	0.000
AFP	0.574	0.333	0.519	0.580	1.162	0.782
Free β-hCG	0.551	0.302	0.540	0.616	1.266	0.672
DD + AFP	0.500	0.079	0.630	0.872	1.841	0.159
DD + free β-hCG	0.500	0.063	0.634	0.895	1.873	0.127
AFP + free β-hCG	0.515	0.349	0.539	0.600	1.264	0.720
DD + AFP + free β-hCG	0.463	0.103	0.642	0.849	1.936	0.192
PE (53)						
DD	0.360	0.151	0.479	0.916	2.357	0.236
AFP	0.184	0.623	0.444	0.771	2.053	0.763
Free β-hCG	0.029	0.887	0.600	0.737	3.849	0.914
DD + AFP	0.382	0.132	0.469	0.923	2.270	0.214
DD + free β-hCG	0.353	0.170	0.478	0.907	2.352	0.262
AFP + free β-hCG	0.662	0.208	0.318	0.807	1.197	0.614
DD + AFP + free β-hCG	0.353	0.151	0.484	0.917	2.406	0.233
SPE (41)						
DD	0.287	0.000	0.512	1.000	3.487	0.000
AFP	0.551	0.146	0.318	0.910	1.548	0.326
Free β-hCG	0.824	0.049	0.258	0.923	1.155	0.276
DD + AFP	0.243	0.000	0.554	1.000	4.121	0.000
DD + free β-hCG	0.235	0.049	0.549	0.981	4.043	0.064
AFP + free β-hCG	0.559	0.122	0.321	0.923	1.571	0.276
DD + AFP + free β-hCG	0.243	0.000	0.554	1.000	4.121	0.000
HDP = GH + PE + SPE (220)						
DD	0.360	0.264	0.768	0.600	2.044	0.412
AFP	0.257	0.605	0.713	0.432	1.537	0.814
Free β-hCG	0.551	0.368	0.650	0.430	1.146	0.821
DD + AFP	0.596	0.068	0.717	0.786	1.565	0.169
DD + free β-hCG	0.603	0.050	0.718	0.831	1.576	0.126
AFP + free β-hCG	0.551	0.332	0.662	0.455	1.212	0.740
DD + AFP + free β-hCG	0.316	0.291	0.784	0.592	2.243	0.425

*D-D* D-dimer, *AFP* alpha-fetoprotein, *free β-hCG* free β subunit of human chorionic gonadotropin, *GH* gestational hypertension, *PE* preeclampsia, *SPE* severe preeclampsia, *FNR* False negative rate, *FPR* False positive rate, *PPV* Positive predictive value, *NPV* Negative predictive value, *+ LR* Positive likelihood ratio, *−LR* Negative likelihood ratio

Evaluation of the diagnostic value of models for GH, PE and SPE showed that the PPV and NPV of DD alone, DD + AFP, DD + free β-hCG, and DD + AFP + free β-hCG were higher than that of AFP + free β-hCG. DD alone in combination with other indicators (AFP alone, free β-hCG alone and AFP + free β-hCG) could reduce FPR and improve + LR.

## Discussion

This research was a retrospective observational case–control study based on prenatal screening in the second trimester. Maternal serum levels of DD, AFP, and free β-hCG in women with HDP (GH, PE, and SPE) and controls were assessed. We established a multi-index model of DD, AFP, and free β-hCG to calculate the risk of HDP. The results demonstrated that levels of DD in the second trimester were significantly higher in the GH, PE, and SPE groups than in the control group (*P* < 0.001). In addition, serum DD levels of pregnant women showed a gradually increasing trend with the progression of HDP, suggesting that DD seems to be the most promising of those markers, with a significant trend related to severity of the disease.

While AFP had diagnostic value for SPE and free β-hCG was not predictive of GH, PE, or SPE, combining free β-hCG and AFP increased the AUC and sensitivity of the prediction model, thereby improving their predictive effects. The best risk calculation model combined DD + AFP + free β-hCG.

For different types of HDP, DD + AFP + free β-hCG had the best diagnostic value for SPE, followed by PE and GH. Therefore, simultaneous screening for fetal aneuploidy and maternal HDP can be performed based on AFP, free β-hCG, and DD in the second trimester of pregnancy. The cut-off values of DD + AFP + free β-hCG for GH, PE, SPE, and HDP were 0.790, 0.952, 0.706, and 1.429, respectively. The positive and negative predictive values of DD + AFP + free β-hCG for GH, PE, SPE, and HDP were 0.642 and 0. 849, 0.484 and 0.917, 0.554 and 1.000, and 0.784 and 0.592, respectively. These results indicated that the appropriate cut-off value differed for different HDP types.

The second trimester maternal weight of women in the GH, PE, and SPE groups was significantly higher than that of the control group (*P* < 0.001), suggesting that increased maternal weight in the second trimester may be connected with HDP. The + LR of DD alone was lower than that of DD + AFP + free β-hCG for PE, SPE, and HDP, indicating that the risk model prediction of DD alone for PE, SPE, and HDP was more likely to be a true positive when the test results were positive.

After analyzing results in Table 3, the most relevant result is that for SPE, DD alone has a sensitivity of 100% with a 0% false negative rate. The addition of the other markers only improve specificity by 4.4% but with a significant increase in economic cost if we want to reply this model in countries without a second trimester Down’s Syndrome screening program. Moreover, DD alone has the high + LR for GH, PE, and SPE (1.478, 2.357, 3.487) considering a good marker above this cut-off. This analysis reinforces the results that DD is possible the best individual predictive marker. In addition, the -LR of DD + AFP + free β-hCG was lower than that of DD alone for PE, suggesting that the risk model prediction of DD alone for PE was more likely to be a true negative when the test results were negative. The -LR of DD alone was lower than that of DD + AFP + free β-hCG for GH and HDP. Therefore, it is necessary to take individual medical measures for the prevention and treatment of HDP.

HDP place an enormous burden on individuals and health care systems worldwide. HDP have a multifaceted symptomatology that ranges in severity from benign disorders to serious multisystem disorders and fetal compromise [26]. It is well-known that stillbirths are closely related to pregnancy complications, and HDP are the most common pregnancy complications [27]. Tao et al. showed that the adjusted risk ratio for a stillbirth for women with HDP compared with normotensive women was 3.1 for singleton pregnancies (95% CI: 2.85–3.37) [28].

Previous research showed a significant elevation of DD in patients with SPE [29]. Activation of the fibrinolytic, inflammatory, and coagulation systems is associated with the occurrence of PE and the triggering of multi systemic compromise, including hypertensive crisis, renal or hepatic dysfunction, and pulmonary edema [16]. Our study demonstrated that combing DD with other screening markers in prediction models improved its diagnostic accuracy and reduced NPR. DD has a high NPV, close to 99%, and can be used as a diagnostic and monitoring tool for disseminated intravascular coagulation and thrombotic complications in patients with malignant neoplasms, PE, migraine, or placental abruption [13, 30, 31].

However, there have been few studies using a DD + AFP + free β-hCG model to predict HDP in the second trimester. Baboolall et al. showed that the AUC of third trimester DD for SPE was 0.828, proving its predictive value for SPE [12]. The results of our study showed that the AUC values of second trimester DD for GH, PE, and SPE were 0.642, 0.767, and 0.905, respectively, and the AUC values of DD for GH, PE, and SPE were all higher than our previous results for third trimester DD (0.560, 0.614, and 0.640, respectively) [32]. Marcq et al. found that the AUC value for the ROC curve of single-indicator plasma DD for the diagnosis of PE was 0.901, and the predicted sensitivity and specificity for PE were 0.962 and 0.714, respectively, which was similar to the results of our study [33].

Though the AUC value for DD combined with AFP + free β-hCG for prediction of PE was 0.788, with a sensitivity of 0.849 and a specificity of 0.647. Pregnancy leads to a gradual increase in circulating DD, and large-scale management studies are needed to determine new thresholds for DD to establish specific reference intervals [34, 35]. However, more attention has been focused on optimizing body mass index (BMI) values during gestation for maternal and neonatal needs [36].

As shown in Table 1, the second trimester maternal weights of the GH, PE, and SPE groups were significantly higher than that of the control group (*P* < 0.001), indicating that pregnant women who gained weight during pregnancy were more likely to develop HDP. Our multivariate logistic regression analysis identified maternal weight as a risk factor for GH, PE and SPE, with ORs of 1.103, 1.093, and 1.080, respectively (*P* < 0.001). However, their relative effects were limited (the 95% CIs for OR ranged from 1.000 to 1.100). Moreover, all MoM values used in this study were corrected by gestational age and maternal weight. Consequently, maternal weight had little effect on predictive ability when using AFP and free-β hCG [24]. The further studies should seek to weight-match control and hypertensive subjects. Rnaghi et al. showed that, compared with women with normal BMI, the incidence of PE may be increased in obese women (odds ratio = 2.36, 95% CI: 1.20–4.65) [37]. A previous meta-analysis showed that increased maternal pre-pregnancy BMI and gestational weight gain were associated with a higher risk of HDP (odds ratio = 2.51, 95% CI: 2.31–2.74) [38]. These results are consistent with our study.

This study support the validity of a strategy for HDP screening that is comparable to and can be performed simultaneously with traditional prenatal screening, requiring only that a blood sample be obtained for AFP and free β-hCG between 15 and 20 weeks 6 days. Early prenatal screening in the second trimester allows for early decision-making and early clinical interventions for pregnant women. The prenatal screening laboratory already detect these markers (AFP and free β-hCG) as part of routine antenatal screening for chromosomal and neural tube defects, so it does not add to overall healthcare cost, yet it might provide prognostic information about potential development of HDP in the pregnancy. The DD would be the only increase needed that would be needed since this is not currently ordered as part of any antenatal screening, if this were to be eventually implemented into clinical practice.

For PE prophylaxis initiated, women with the high-risk factors for PE should receive low-dose aspirin between 12 and 28 weeks of gestation, optimally before 16 weeks, and continuing until delivery [39]. By broadening prenatal screening to include pregnancy complications such as PE, the risk for HDP can quickly be calculated in the second trimester. Interventions, such as providing low-dose aspirin, can be initiated at the same time to reduce the rate of birth defects and perinatal maternal death with minimal interruption to the patient [40–42]. Considering a second trimester screening with free β-hCG and AFP could affect the external validation of results, because those markers are not universally available in all countries. DD is an interesting marker for PE, as an expression of vascular dysfunction and a pro-coagulant state commonly observed in patients with PE. As the patients are older, the FPR of the DD levels are increasing which reduced the specificity of the test in the patients. The FPR can be reduced by using an age-adjusted DD threshold which is calculated as the age of patients over 50 years of age multiplied by 10 ng/mL [43]. In addition, the pregnant women in this study were all around 28 years old, which the impact of age was negligible.

We acknowledge several limitations of the study. First, the lack of blinding of patients and providers may have introduced bias as well as the early timing associated with screening, as early diagnosis of GH is frequently conflated with chronic hypertension while the symptoms of SPE are overlooked. Second, this was a retrospective, hospital-based, single-center study. Therefore, a causal relationship between DD and HDP could not be assessed. Retrospective designs have overestimated prediction, and the + LR have reduced when used in a case–control study. Third, the relatively fewer cases compared with big data in the control group may have affected the conversion of DD concentrations to MoM values and influencing modeling results. Forth, more demographical characteristics should be included, such as chronic hypertension, smoking habit, autoimmune diseases and perinatal outcomes (birth weight percentile, gestational age at delivery, csection rate, admission), et.al.

Our project added second trimester DD levels to prenatal screening and used PerkinElmer’s Lifecycle 4.0 software to calculate the risk of HDP to explore whether a combined DD screening regimen was superior to the traditional screening regimen. A combination of prenatal screening markers in the second trimester was superior to traditional prenatal screening for GH, PE, and SPE, especially when the risk model was constructed with DD, AFP, and free β-hCG. This method also allows for targeted and personalized prevention and treatment of HDP.

## Conclusions

In conclusion, based on AFP, free β-hCG, and DD in the second trimester of pregnancy, fetal aneuploidy screening and maternal HDP prediction can be performed simultaneously. In addition, DD alone is an efficiency individual predictive marker for predicting HDP. The results showed that the risk prediction model constructed with DD + AFP + free β-hCG and DD alone had better screening efficiency compared with the original model of AFP + free β-hCG. DD alone and DD + free β-hCG + AFP had the best diagnostic value for SPE, followed by PE and GH, with different predictive values for different types of HDP. The ROC of our study demonstrates > 0.800 for predicting PE, SPE, and combined HDP using a combination of DD, AFP, and free β-hCG. These findings bear potential clinical importance, as may prove useful in screening women for future development of HDP. The risk prediction model can improve the sensitivity of HDP screening and provide new screening methods for clinical treatment of HDP. To adapt to the health care needs of high-risk pregnant women, health providers should consider more widespread application of available methods and innovative prenatal screening programs that facilitate early interventions.

## Data Availability

Data sharing is not applicable to this article as no new data were created or analyzed in this study.

## Abbreviations

DD: D-dimer
free β-hCG: Free beta subumit of human chorionic gonadotropin
HDP: Hypertensive disorders of pregnancy
GH: Gestational hypertension
PE: Preeclampsia
SPE: Severe preeclampsia
MoM: Multiple of the median
ROC: Receiver operating characteristic
AUC: Area under curve
PPV: Positive predictive value
NPV: Negative predictive value
+ LR: Positive likelihood ratio
−LR: Negative likelihood ratio
FNR: False negative rate
FPR: False positive rate
